# Supplementary figures and images for: The Sleep Quality- and Myopia-Linked PDE11A-Y727C Variant Impacts Neural Physiology by Reducing Catalytic Activity and Altering Subcellular Compartmentalization of the Enzyme
Source: Cells. 2023 Dec 14;12(24):2839. doi: 10.3390/cells12242839 (PMC10742168; doi:10.3390/cells12242839)

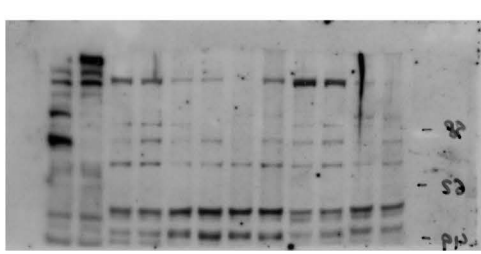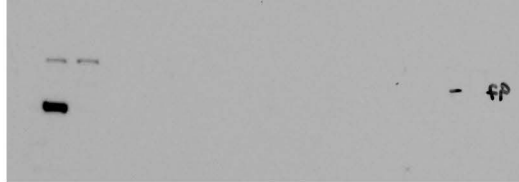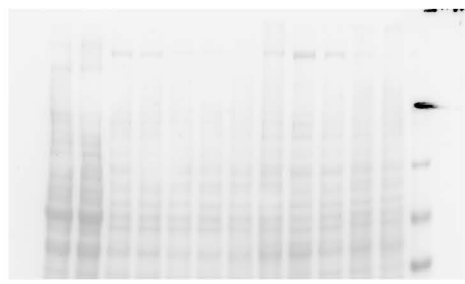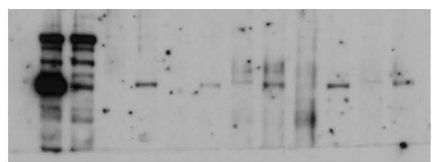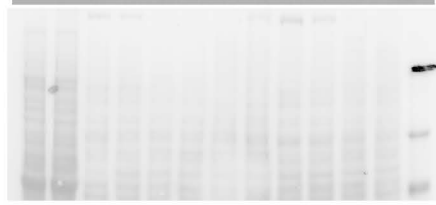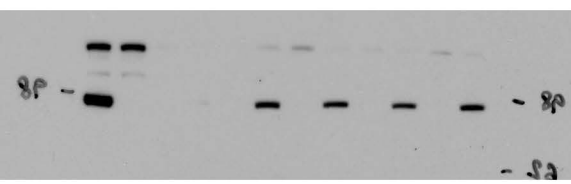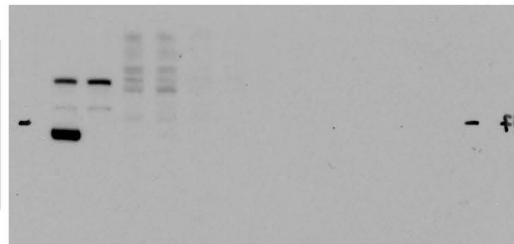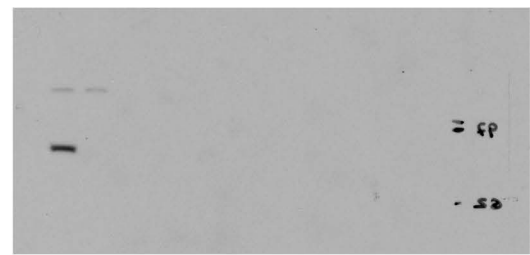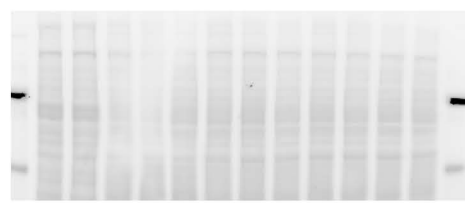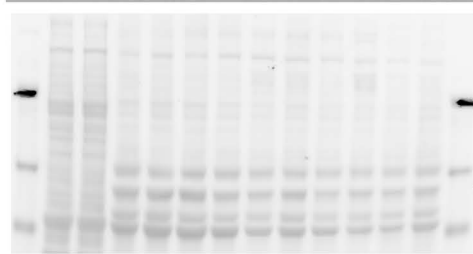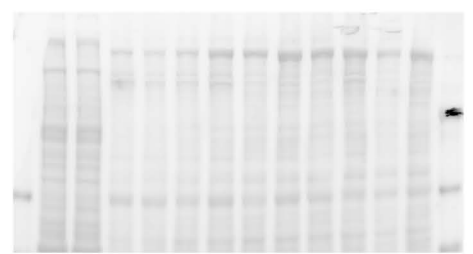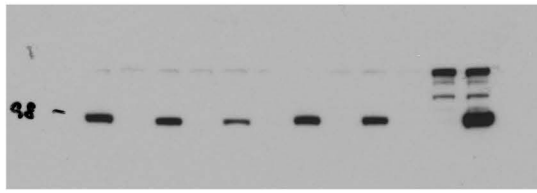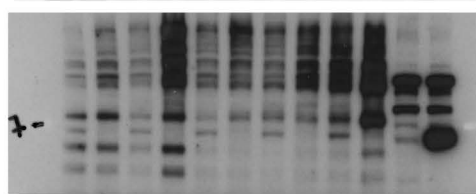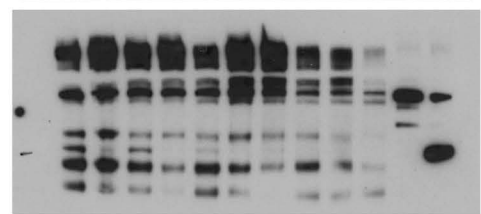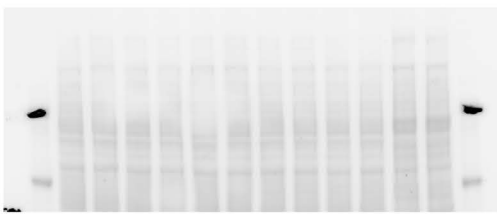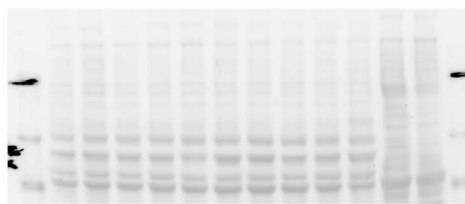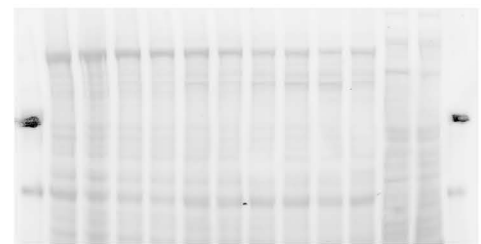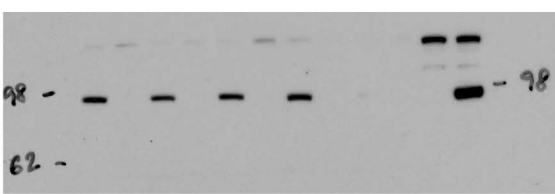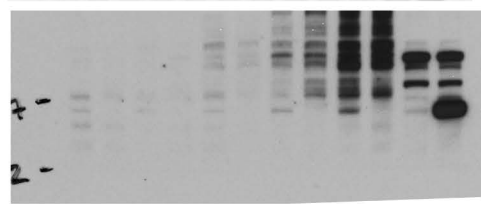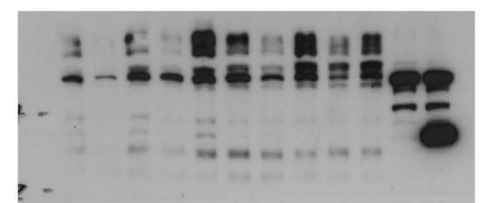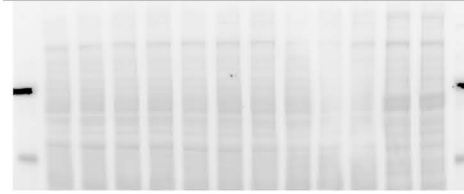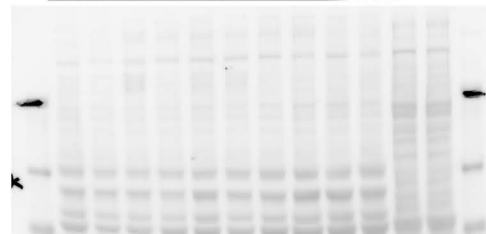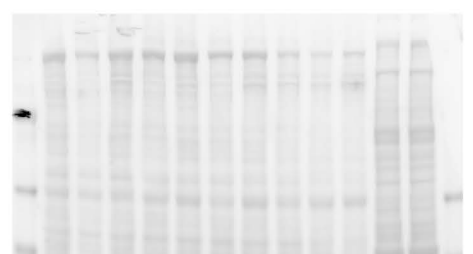

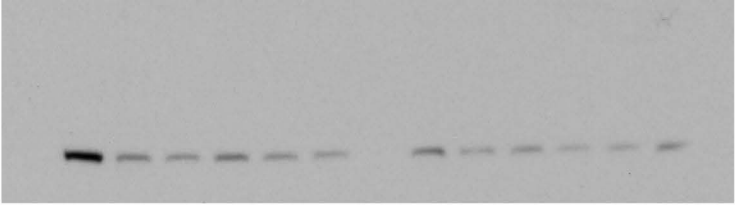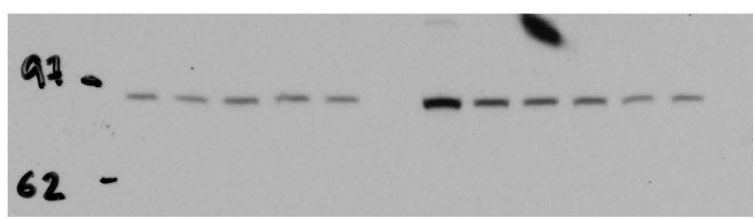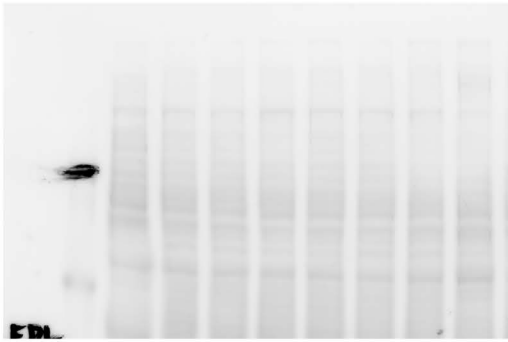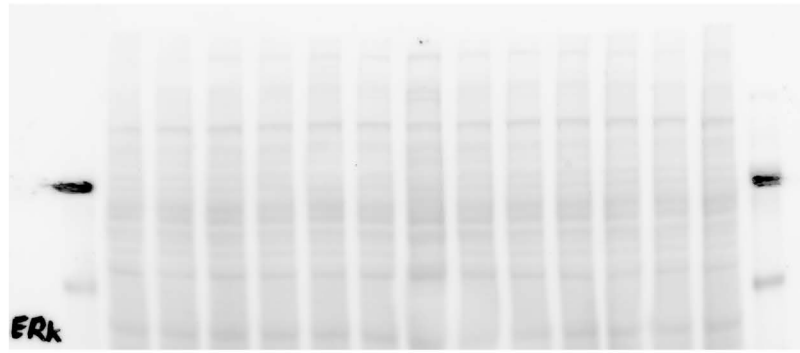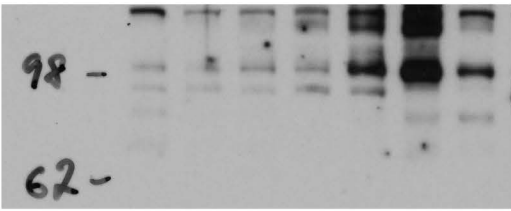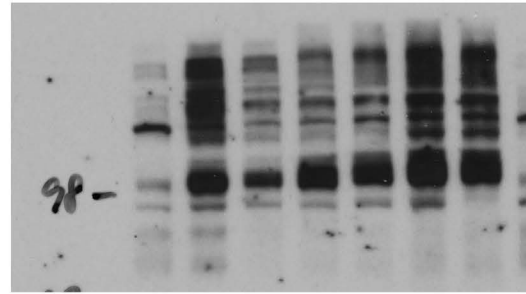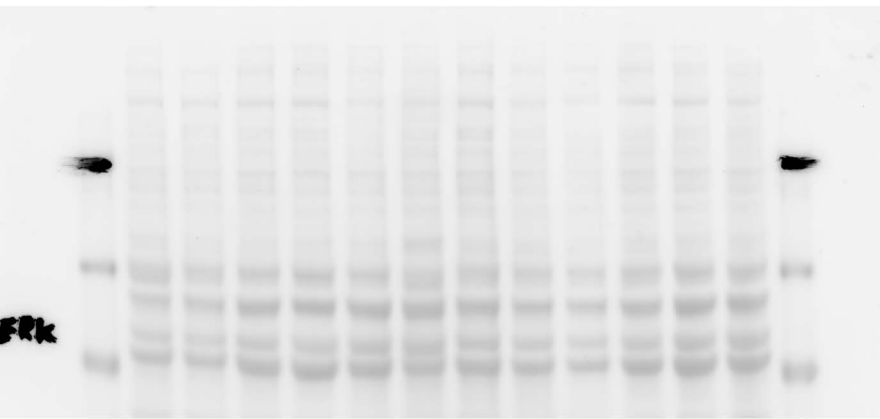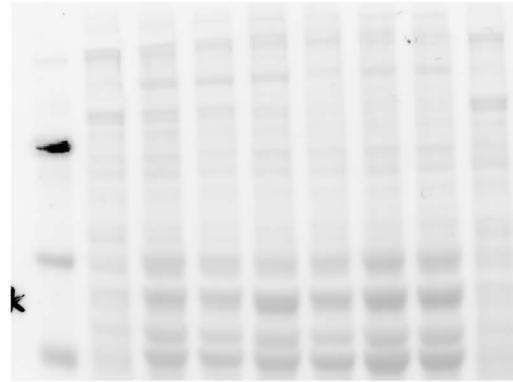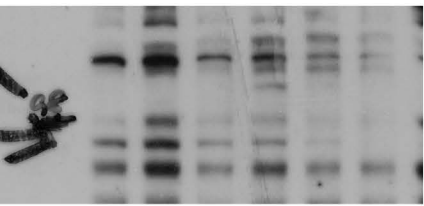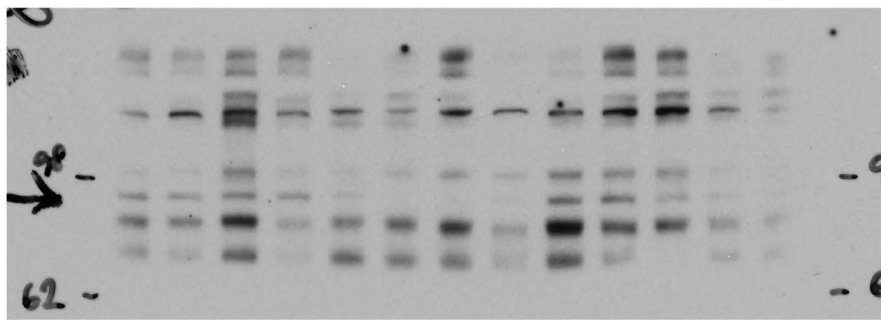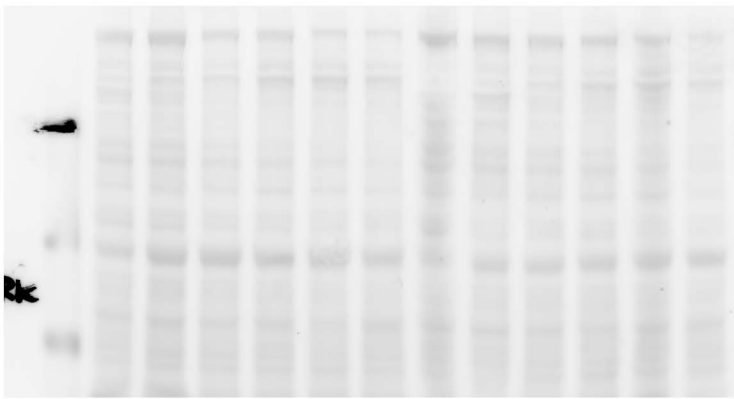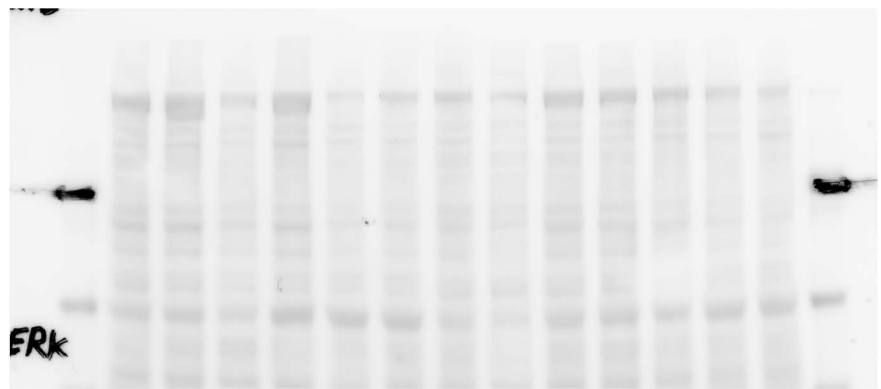

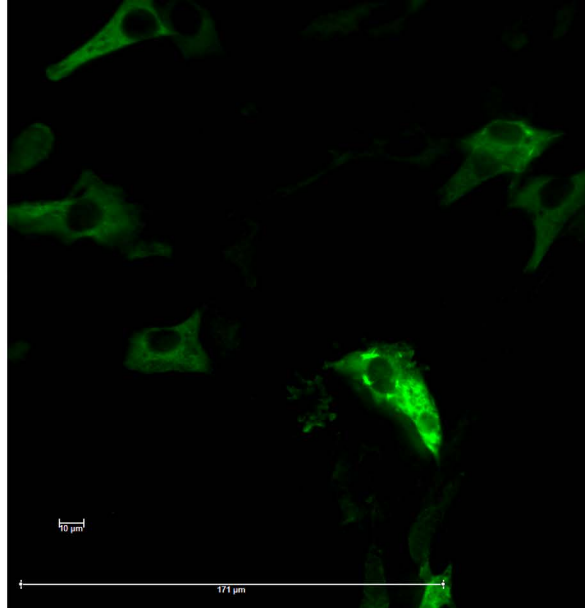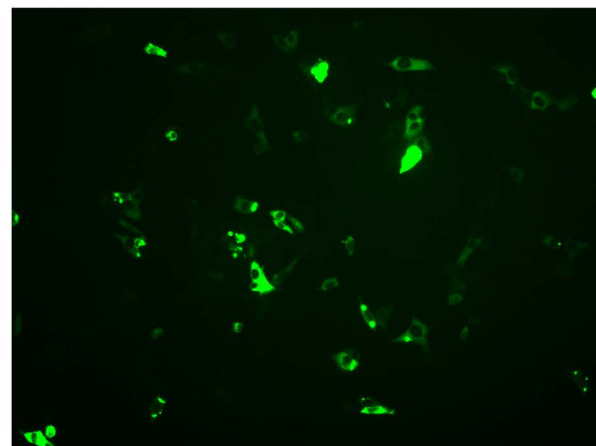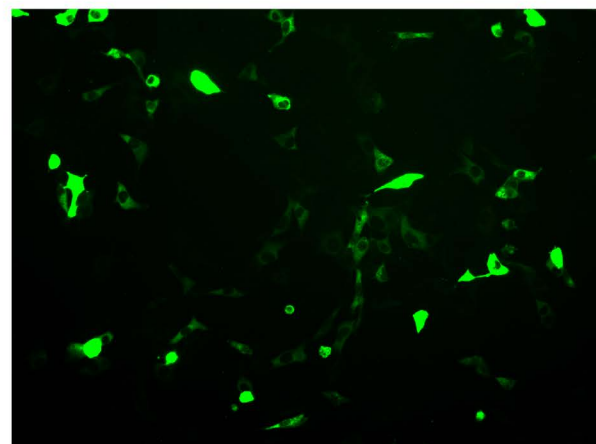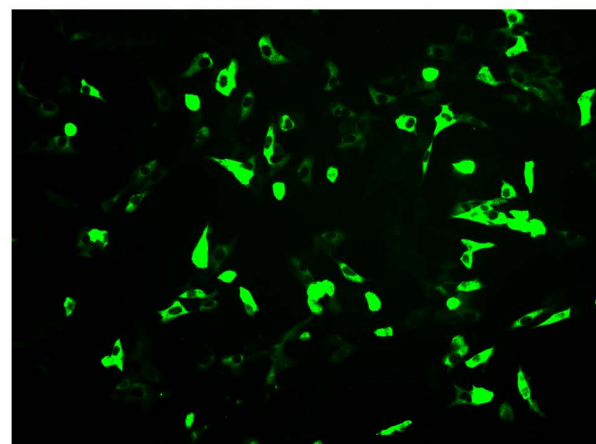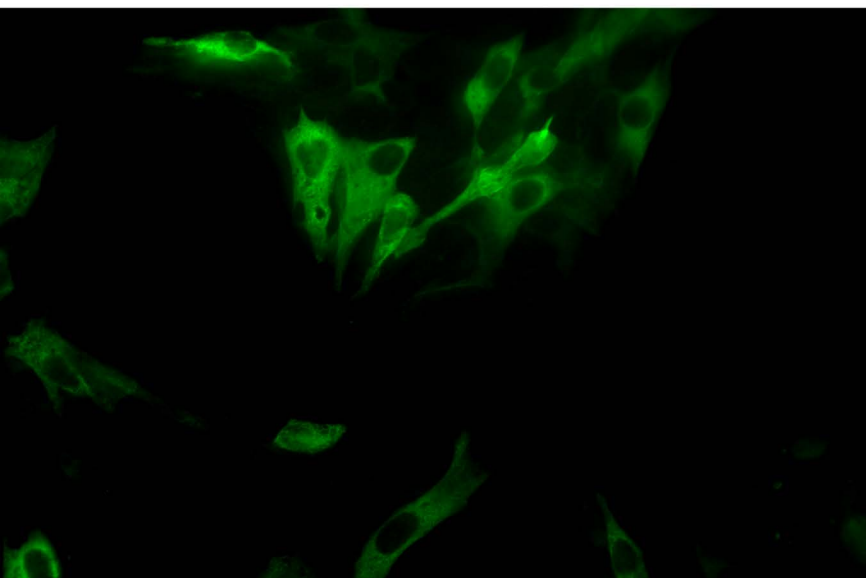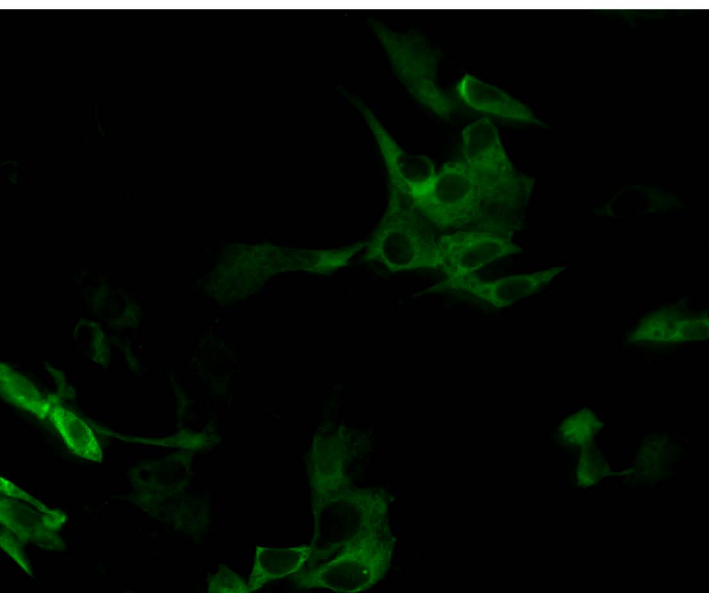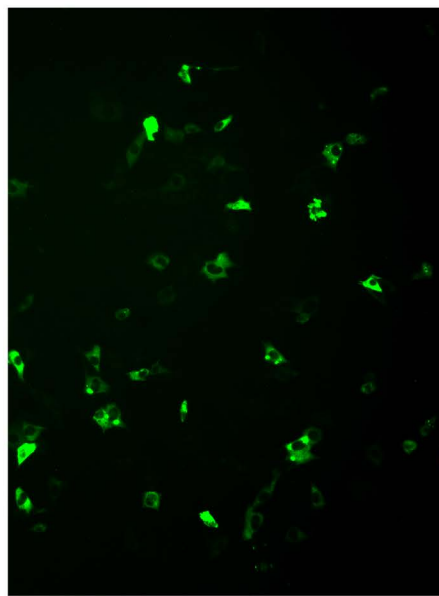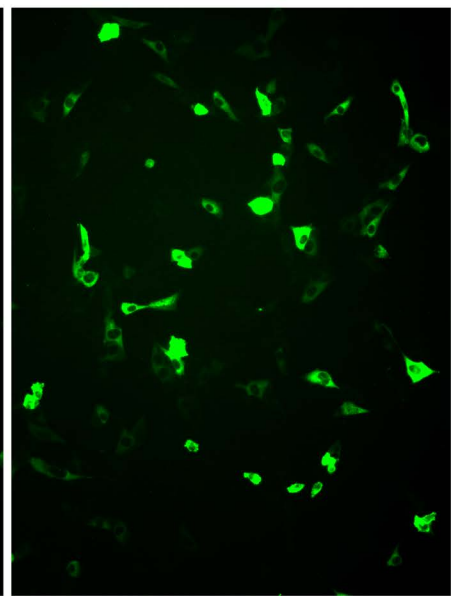

★

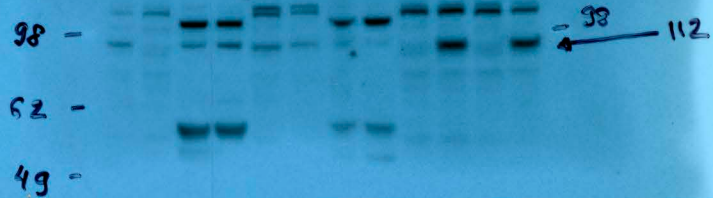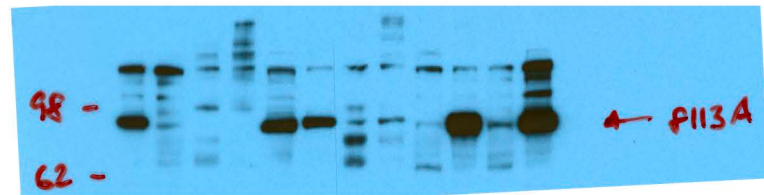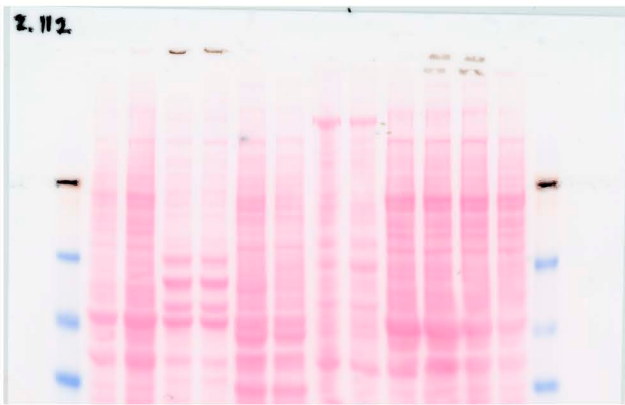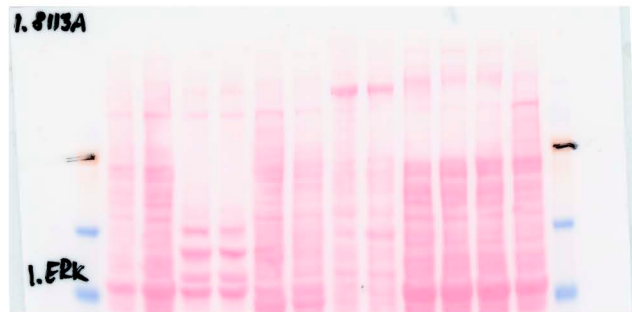

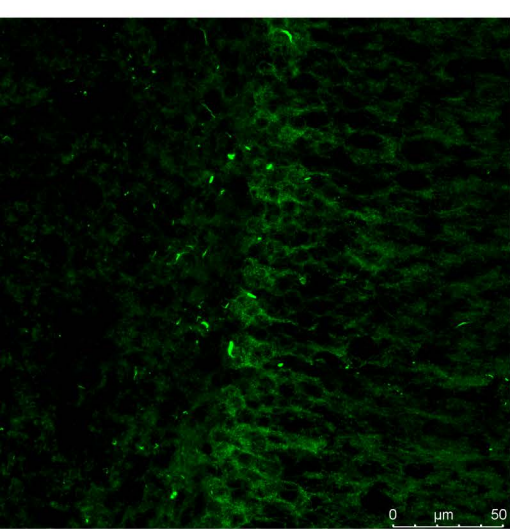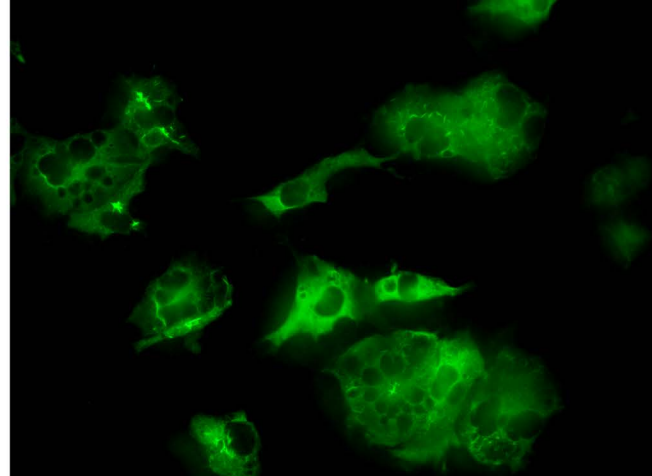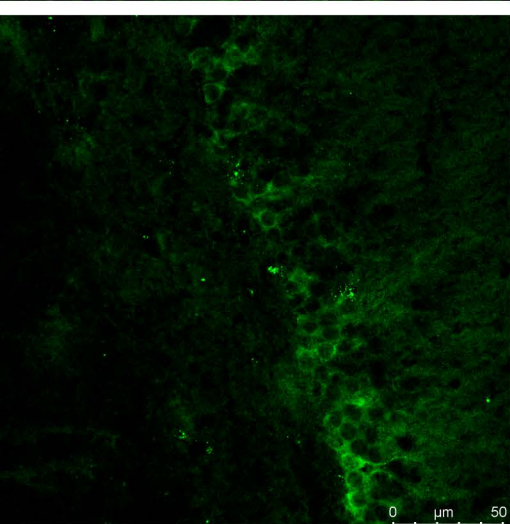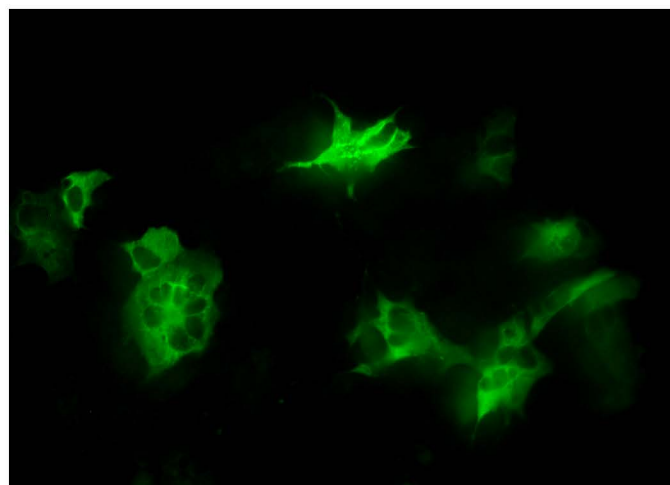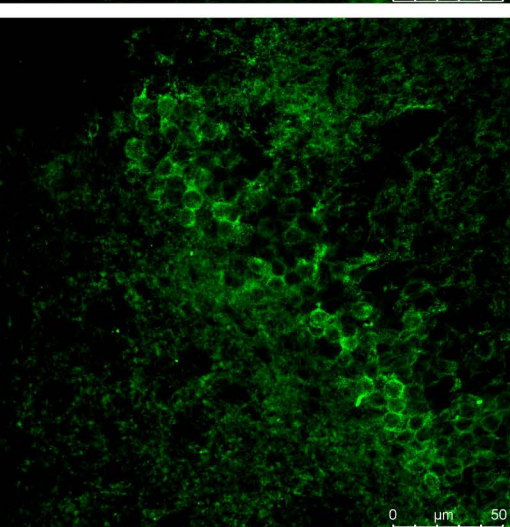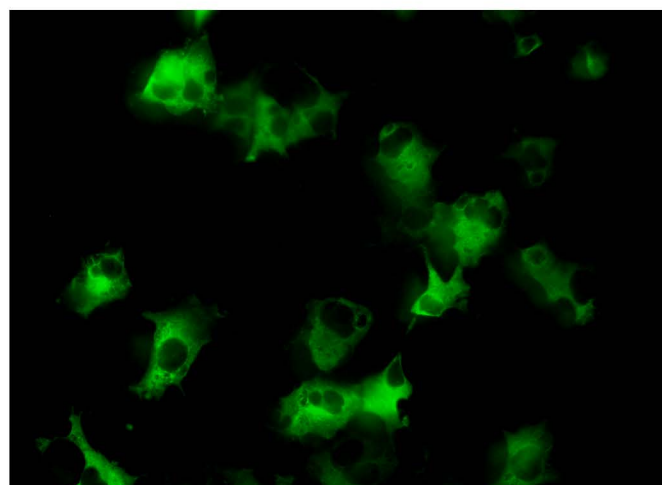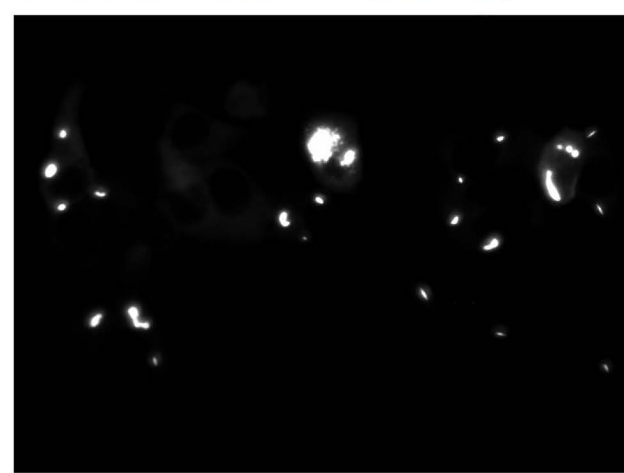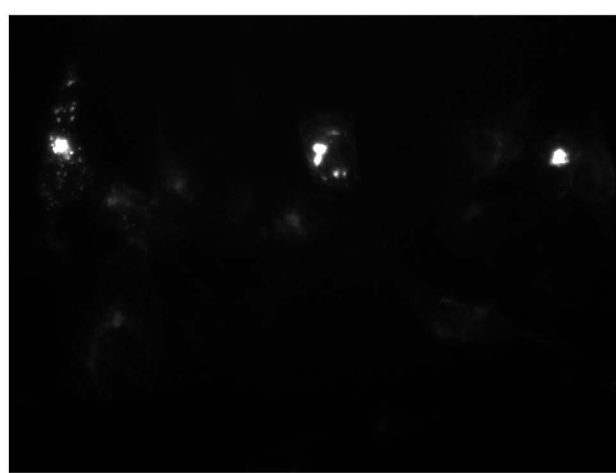

Supplement: Supplementary file 1 [file cells-12-02839-s001.zip › Sbornova et al UncroppedImages.pdf]
